# Cross-cultural measurement invariance evidence of individualism and collectivism: from the idiosyncratic to universal

**DOI:** 10.3389/fpsyg.2023.1150757

**Published:** 2023-09-27

**Authors:** Francisco Leonardo Soler-Anguiano, Sofía Rivera-Aragón, Rolando Díaz-Loving

**Affiliations:** Faculty of Psychology, National Autonomous University of Mexico, México City, Mexico

**Keywords:** psychometric properties evidence, culture, propensity score matching, scale development, Mexico, United States

## Abstract

**Introduction:**

Culture plays a fundamental role in shaping human behavior, with individualism and collectivism being key cultural dimensions. However, existing scales for measuring these constructs, such as the INDCOL scale, have demonstrated issues when applied in diverse cultural contexts. To address this, we present the translation and adaptation of the Mexican Vertical and Horizontal Individualism and Collectivism Scale (MXINDCOL) into English, aiming to identify both universal and culture-specific elements.

**Methods:**

Data were collected from 1124 participants (371 from the United States, 753 from Mexico) using the MXINDCOL and INDCOL scales. Propensity score matching was applied to balance demographic differences between the samples. Confirmatory Factor Analysis (CFA) assessed model fit, and cross-cultural measurement invariance was examined. Reliability, convergent and discriminant validity were also assessed.

**Results:**

The English-translated MXINDCOL scale demonstrated good model fit in both US and Mexican samples, outperforming the INDCOL scale. Reliability values were higher for the MXINDCOL scale compared to INDCOL. Cross-cultural measurement invariance was established, allowing for meaningful comparisons between the two cultures. US participants scored higher on vertical collectivism, while Mexican participants scored higher on horizontal collectivism and horizontal individualism.

**Discussion:**

The MXINDCOL scale offers a culturally sensitive measurement of individualism and collectivism, addressing issues found in existing scales. It provides a more accurate assessment of cultural orientations and enriches the understanding of cultural dimensions by incorporating idiosyncratic elements. Further research in diverse cultural contexts is recommended to validate and refine the scale, contributing to a more nuanced understanding of cultural variations in individualism and collectivism.

## 1. Introduction

Culture has been understood as collective cultural programming, where actions, feelings, and thoughts differ between groups (Hofstede, 2001); it highlights the adaptive role that society transmits from one generation to the next through a system of shared meanings (Triandis, 1994, 2000, 2001; Singelis et al., 1995). To understand how this cultural orientation interacts and describes cultures, it is essential to have evidence regarding reliable and valid ways to assess the construct in different contexts. When assessing the constructs of individualism and collectivism, some issues have been observed with the widely applied individualism and collectivism scale (INDCOL; Triandis and Gelfand, 1998). Some studies have found a lack of adequate psychometric properties when the scale is translated and applied in non-English-speaking contexts (e.g., Díaz Rivera et al., 2017), and some others have re-specified the model to reach a valid and reliable version (e.g., Li and Aksoy, 2007; Germani et al., 2019). Conversely, researchers such as Díaz-Loving et al. (2018) have created a scale in a Spanish-speaking context, the Mexican Vertical and Horizontal Individualism and Collectivism Scale (MXINDCOL), showing adequate psychometric properties. Although this scale has shown good psychometric properties and model fit, it has not been adapted, translated, and tested in different contexts. Hence, we present the translation and adaptation of the MXINDCOL into English.

Cultural orientations, encapsulated by the concept of individualism and collectivism, play a pivotal role in shaping behaviors and societal dynamics. Hofstede (2001), in an attempt to understand the differences derived from cultural characteristics, categorized general cultural dimensions as power distance, uncertainty avoidance, masculinity/femininity, and individualism–collectivism. Of the various dimensions, individualism–collectivism has garnered significant attention as a widely explored and consistently relevant factor in delineating cultural characteristics. In this manner, cultural influences shape our focus, leading us to emphasize individual traits and their internal aspects (such as attitudes and beliefs) when displaying higher individualistic tendencies while highlighting the significance of group dynamics, roles, norms, duties, and interpersonal bonds within the group when exhibiting stronger collectivist orientations (Triandis, 2000). In cultures with deep-rooted collectivist values, exemplified in regions like Asia and Latin America, where societies are relatively homogeneous and densely populated, individuals are more inclined to demonstrate interdependence, considering themselves interconnected with others from the group. Groups, such as family and co-workers, prioritize group goals (Triandis, 2001), where decisions impact others and both positive and negative results are shared (Chen and West, 2008). Thus, despite being societies that are focused on the interdependence between individuals, there is a stark contrast in the definition of groups, such as the close group and the foreign group (Triandis, 2006), with a general global pressure to conform to the group (Singelis et al., 1995). This type of society must also be organized and adhere to many behavioral rules among community members (Triandis, 2000). It is characterized by a high attachment to values such as patriotism, heroism, loyalty, and self-sacrifice (Triandis, 2006).

Conversely, in individualistic societies, individuals see themselves as autonomous, independent of their groups, prioritizing their personal goals over those of their group, and behaving primarily based on their attitudes rather than the norms of their group (Markus and Kitayama, 1991a, 1991b), emphasizing personal values and prioritizing beliefs over norms (Triandis, 2006). Thus, individualism is formed by independence, uniqueness, and competitiveness (Chen and West, 2008). In other words, as Singelis et al. (1995) and Triandis (2000, 2011) mentioned, collectivists focus mainly on relationships with others, preferring methods of conflict resolution that do not destroy relationships, while individualists focus on achieving justice and being willing to face disputes to resolve conflicts. Autonomy has been previously regarded as a trait inherent to individualists. However, Chen and West (2008) demonstrated that humans require autonomy and interdependence. This suggests that autonomy and collectivism are not inherently contradictory. Consequently, autonomy holds a significant presence among both individualists and collectivists.

The polarized dichotomy of this construct has been deemed unproductive in the field of cultural comprehension (Comadena et al., 1998; Omi, 2012). The evidence demonstrates a stance favoring adopting a multidimensional approach to attain a deeper understanding of the concepts of individualism and collectivism (Schwartz, 1990). Shulruf et al. (2007) extended this concept by considering dimensions such as responsibility, uniqueness, competitiveness, advice, and harmony, which were grouped into 2 s-order factors representing individualism and collectivism. They assessed these dimensions using the Auckland Individualism and Collectivism Scale. While the Auckland Individualism and Collectivism Scale (Shulruf et al., 2007) exhibited improvement compared to the two-factor scale, its model fit ranged from acceptable to mediocre (Shulruf et al., 2007).

Likewise, complements of cultural emphasis based on hierarchy and equality have shown a better adjustment (e.g., Oyserman et al., 2002; Shavitt et al., 2011). Specifically, Singelis et al. (1995) have sought to complement the construct by incorporating elements of verticality and horizontality. Verticality, on the one hand, pertains to hierarchical dimensions within interpersonal relationships. It entails sustaining subordinating relationships between the individual and the collective (Chen et al., 1997). Consequently, collectivist individuals embracing a vertical orientation become embedded in this cultural paradigm. These individuals perceive themselves as complementary constituents of the group, where each member holds distinct traits, and certain members wield higher status than others (Triandis, 1995). The self-concept of these vertically-oriented collectivists is interconnected and distinct from that of others, a feature fostering the endorsement of inequality as a favorable norm, wherein serving and sacrificing for the group remain pivotal. Essentially, they reflect conformity to the authorities (Bond and Smith, 1996), making them more likely to do what is expected (Singelis et al., 1995). On the other hand, according to Singelis et al. (1995), vertical individualism is a cultural pattern in which the autonomous self is postulated. Still, individuals see each other as different and with high inequality. The self is independent and separate from the self of others, and competition and self-sufficiency are essential aspects of this pattern. This vertical aspect of individualism that acknowledges inequalities between people requires a level of conformity to hierarchical service.

Instead, horizontality reflects equality in culture and seeks similarity between individuals (Triandis, 2000), increasing the sense that people should be free from the influence of others (Singelis et al., 1995). Therefore, horizontal collectivism defines a cultural pattern in which the individual sees himself as an aspect of the group, where everyone is similar to each other, the self is interdependent, and at the same time, the self will seek to complement the self of others. Thus, equality will be the essence of this pattern (Triandis, 1995). Alternatively, horizontal individualism is a cultural pattern where the autonomous self is postulated. Nevertheless, the individual is essentially the same as the others, with independence from other individuals being the differential characteristic (Singelis et al., 1995).

The main challenge in studying individualism and collectivism is to identify evidence for reliable and valid ways to assess the construct. The INDCOL scale adapted by Triandis and Singelis (1998) found a strong association between subjective individualism and being young, traveling, valuing privacy, and having an occupation that allows one to make their own decisions while ignoring the needs of others. Conversely, subjective collectivism involves choosing family goals over personal ones, feeling close to the group, living in a small community, being strongly influenced by the traditions of parents and grandparents, and being interdependent on finances. It also involves having had a traditional formal upbringing, growing up in extended families, having a job that requires speaking from the point of view of others, needing to be around others for fun, and having an occupation that requires focusing on the needs of others.

The problem arises when the measurements are used in regions or cultures other than those for which they were created. The proof of this problem was found when psychometric properties of different adaptations of the INDCOL were assessed when applied in a different cultural context than intended. Biddlestone et al. (2020) showed reliabilities below 0.69 in a sample majority of British and US people. Li and Aksoy (2007) showed a poor model fit in a US and Turkish sample regarding the original structure. Owing to that, the authors decided to set a new model where the item “*To me, pleasure is spending time with others,”* originally established in the horizontal collectivism dimension, was better fitted in the vertical collectivism dimension. Simultaneously, two items were removed to achieve a better model fit; one from horizontal collectivism and another from horizontal individualism. With this modification, the model fit showed a fair improvement over the original configuration proposed by Triandis and Gelfand (1998; see Li and Aksoy, 2007). In the Italian context, Germani et al. (2019) have supported the valid and reliable Triandis and Gelfand (1998) version with the re-specified model proposed by Li and Aksoy (2007). In Mexico, the scale was back-translated following the Brislin (1970) method. From this, it was applied to a Mexican sample to identify the psychometric properties of the back-translated INDCOL scale. With this, the same four factors identified by Triandis and Gelfand (1998) were identified only after some items were dropped (see Díaz Rivera et al., 2017). In Díaz Rivera et al. (2017) study, the resulted factor composition showed low reliability ranging from 0.51 to 0.69. Germani et al. (2019), Li and Aksoy (2007), as well as Díaz Rivera et al. (2017), have emphasized that through item re-specification and the removal of certain items, a better fit with the construct of individualism and collectivism in a different culture can be achieved. However, it is worth considering that solely eliminating items may prove insufficient when conducting cross-cultural comparisons and measurements. This is because idiosyncratic items from the culture of origin might not hold relevance in translation, potentially resulting in the retention of only universal items in the translated version. Such procedures could consequently reveal only those elements that are universally shared between cultures while discarding those that are culture-specific.

The assessment field has long been influenced by an ethnocentric perspective, where one’s group standards are often applied as a generalized perspective for evaluating other groups’ standards (Berry et al., 2011). For instance, Western societies may view a firm handshake and coordinated eye contact as appropriate, whereas in other parts of the world, a bow without direct eye contact holds cultural significance. However, it is crucial to mitigate these ethnocentric judgments in the realm of cross-cultural psychology. Such judgments can lead to misinterpretations of behaviors in different cultural contexts. Furthermore, ethnocentrism can seep into cross-cultural inquiries, potentially skewing research outcomes. To address this issue and avoid fixating on a single cultural perspective, Campbell (1970) proposed a valuable solution: conducting studies on a phenomenon in a culture different from the one of its origin. This approach allows researchers to discern differences stemming from ethnocentric bias from those arising due to genuine variations between cultures. It aligns with the indigenous conception, emphasizing the significance of construct conceptualization and operationalization in uncovering and assessing the universal and specific aspects of a perspective when studying phenomena across diverse regions and cultures (Díaz-Loving, 1998).

An underlying issue when analyzing the INDCOL scale comes from identifying a mix of orientations in terms of construct focus operationalization. From the narrative, it is clear that the individualism and collectivism scales (Triandis and Gelfand, 1998) are focused on the subjective measurement of the cultural syndrome. Yet, it might present some biases regarding the items (*cf.* Van de Vijver and Poortinga, 1997). These biases can lead to a non-unified pool of items according to the construct domain. On the one hand, some items try to assess a personality trait related to the construct, like “*I often do…”* or “*If …, I would feel …,”* where the self-perspective comes afloat as the main measurement domain. On the other hand, other items are focused on assessing norms and beliefs at the individual level, with items like, “*Parents and children must* …” or “*Competition is the* …,” where the norm perspective of *must* and *have to be* orientations are the main measurement domains (See Appendix).

Having this in mind, Soler-Anguiano and Díaz-Loving (2017) elaborated a scale by creating original items based on the literature and extracting the conceptual definitions of the construct and the dimensions. The items were made by prioritizing local conceptualizations of the universal psychological phenomenon, which is typically called a bottom-up approach (see Cheung et al., 2011) that provides new elements to distinguish between the universal and cultural variables in the construct. The process of scale creation in Mexico was specifically targeted at the individual level of the construct, encompassing aspects such as personality traits. This approach diverged from the combination of norms, beliefs, and personality traits observed in the INDCOL (Triandis and Gelfand, 1998). They also identified and included elements that are appropriate to the Spanish language. The result of the exploratory factor analysis replicated the four factors with adequate psychometric properties, where the reliabilities ranged from 0.62 to 0.84. Despite having adequate psychometric properties, there could also be culturally specific elements that might complement the general theoretical postulates. From this, Díaz-Loving et al. (2018) extracted and incorporated these elements with which they created a valid and reliable scale for Mexicans. On the one hand, incorporating the items “*I give myself to others without expecting anything in return,”* and “*I collaborate with others to make things work out well”* in the horizontal collectivism dimension; the items reflect idiosyncratic elements of collectivist cultures regarding doing everything for the group and not just feeling good for others (Triandis, 2000, 2001, 2006; Chen and West, 2008), elements missed in the Triandis and Gelfand (1998) scale. On the other hand, for the vertical collectivism dimension, the item “*I prefer not saying anything over making others feel uncomfortable”* was included to reflect abnegation, typically identified in vertical cultures like Mexico, where reference group members’ well-being is more important than oneself. For instance, a mother is the most valuable group member in the Mexican culture (Díaz-Guerrero, 1994). The MXINDCOL showed internal consistency indices ranging from 0.61 to 0.77 in a sample of participants from four regions of Mexico (Díaz-Loving et al., 2018). Soler-Anguiano et al. (2023) showed internal consistency indices below 0.85 in another sample of Mexican participants. Additionally, Díaz-Loving et al. (2018) confirmed the factorial structure identified, finding a good fit [χ*^2^* (98) = 198.35, CFI =0.94, GFI =0.95, RMSEA =0.046]. It is also shown that the MXINDCOL presents measurement invariance within three regions of Mexico (Díaz-Loving et al., 2018). The confirmatory factorial analysis and the measurement invariance analysis reflect its ideal application in the Mexican population.

Hence, it is advisable to incorporate culture-specific components into scales to effectively capture the construct in diverse contexts. Therefore, the primary aim of the current study was to adapt and translate the MXINDCOL, with the goal of identifying both universal and culture-specific elements. Rather than solely presenting an adapted and translated version of a scale originally developed in a different context, this study aimed to provide a comprehensive analysis. By comparing the psychometric properties and model fit of the MXINDCOL with the widely used INDCOL scale, this study also seeks to discern the strengths and weaknesses of each scale in capturing cultural orientations. Furthermore, the study endeavors to assess cross-cultural measurement invariance across a US and Mexican sample, shedding light on the extent to which these scales perform consistently across diverse cultural contexts.

The hypotheses underpinning the aims of this study are rooted in the belief that the MXINDCOL scale, a culturally sensitive adaptation, will outperform the widely used INDCOL scale in capturing the complexities of individualism and collectivism across different cultural contexts. We hypothesize that the MXINDCOL scale, with its focus on individual-level personality traits and its alignment with local conceptualizations, will demonstrate superior psychometric properties and model fit compared to the INDCOL scale when applied to both US and Mexican samples. Furthermore, we anticipate that the MXINDCOL scale will exhibit cross-cultural measurement invariance, indicating its applicability and consistency across diverse cultural backgrounds. Overall, our study’s hypotheses revolve around the notion that cultural specificity and precision in scale construction, as exemplified by the MXINDCOL, will lead to more accurate assessments of the nuanced variations in individualism and collectivism, ultimately contributing to a more refined understanding of these constructs in different cultural settings.

## 2. Materials and methods

### 2.1. Participants

The study used a cross-sectional design. Prolific Academic was used to collect data from the US participants, who each received £2 (for an approximately 20-min survey). Furthermore, undergraduate and graduate students collected data for the Mexican sample and received course credits. The platform Prolific Academic is a crowdsourcing research platform in which participants are more diverse and more representative, resulting less dishonest response rates compared with other platforms (Peer et al., 2017). There is also evidence that crowdsourced convenience samples and undergraduate convenience samples provide results that have been replicated with representative samples (Krupnikov et al., 2021).

The study was conducted entirely online. The total sample consisted of 1,124 participants: 371 from the United States and 753 from Mexico. If there is an interest in exploring cultural differences, it is important to control for demographic differences across cultural groups to avoid confounders or wrong conclusions (Van de Vijver and Leung, 2021). A preliminary inspection of the sample revealed an unequal distribution of the demographic characteristics of participants (Table 1). Thus, a propensity score matching method was used to counterbalance possible confounding effects (Rosenbaum and Rubin, 1983, 1985). The idea of the procedure is to estimate and match participants to be closely balanced on confounding background characteristics. After the propensity score matching method, the sample was reduced, and cultural differences in demographic characteristics disappeared (Table 1). Therefore, all the analyses were run with these resultant subsamples after propensity score matching.

**Table 1:** Demographic characteristics of participants and group difference effect sizes (φ, Cohen’s d, and Cramer’s V) between the United States and Mexico samples before and after propensity score matching.

Variables	Complete original sample				After propensity score matching			
	United States (*n* = 371)	Mexico (*n* = 753)	Value of *p*	Effect size	United States (*n* = 292)	Mexico (*n* = 332)	Value of *p*	Effect size
Sex: Female – *n* (%)	247 (66.6%)	429 (57%)	< 0.002	*φ* = 0.09	220 (75.3%)	230 (69.3%)	0.09	*φ* = 0.06
Age – *M (SD)*	38.23 (14.74)	30.22 (13.77)	< 0.001	*Cohen’s d* = 0.56	40.04 (13.60)	41.91 (14.12)	0.09	*Cohen’s d* = 0.13
Highest academic degree – *n (%)*			< 0.001	*Cramer’s V* = 0.23			< 0.001	*Cramer’s V* = 0.16
High school studies or lower	153 (41.2%)	439 (58.3%)			92 (31.5%)	107 (32.2%)		
Bachelor studies	155 (41.8%)	281 (37.3%)			138 (47.3%)	193 (58.1%)		
Master studies	47 (12.7%)	27 (3.6%)			46 (15.8%)	26 (7.8%)		
Post-graduate or higher	16 (4.3%)	6 (0.8%)			16 (5.5%)	6 (1.8%)		
Race – *n* (%)								
Asian	56 (15.1%)	0			45 (15.4%)	0		
Biracial/Mixed race	19 (5.1%)	1 (0.1%)			14 (4.8%)	0		
“Black” or African American	27 (7.3%)	0			20 (6.8%)	0		
Hispanic/Latino	34 (9.2%)	749 (99.5%)			23 (7.9%)	332 (100%)		
Middle Eastern/Arab	1 (0.3%)	0			1 (0.3%)	0		
Native Hawaiian or Pacific Islander	2 (0.5%)	0			1 (0.3%)	0		
“White”	231 (62.3%)	3 (0.4%)			187 (64%)	0		

### 2.2. Instruments

The MXINDCOL (Díaz-Loving et al., 2018) comprises 16 items from four dimensions: Horizontal collectivism (e.g., *I give myself to others without expecting anything in return*); horizontal individualism (e.g., *I am original, just as others may be*); vertical collectivism (e.g., *My happiness depends on others’ happiness*); and vertical individualism (e.g., *I enjoy being in situations that involve competing with others*). All questions were answered on a 7-point Likert-type scale from 1 (not at all) to 7 (very much). The items of the MXINDCOL (Díaz-Loving et al., 2018) were back-translated and adapted from Spanish to English by our research team and a Spanish language professional, prioritizing meaning over literality. Finally, refinements and corrections were made according to and in agreement with the authors of the original scale (see Appendix). The English-translated version of the scale was administered in the United States, while the original Spanish version was administered in Mexico.

The INDCOL (Triandis and Gelfand, 1998) comprises 16 items distributed in 4 dimensions: Horizontal individualism (e.g., *I would rather depend on myself than others*); vertical individualism (e.g., *Winning is everything*); horizontal collectivism (e.g., *If a co-worker gets a prize, I would feel proud*); vertical collectivism (e.g., *Parents and children must stay together as much as possible*). All questions were answered on a 5-point Likert-type scale from 1 (definitely no) to 5 (definitely yes). This original version of the scale was administered in the United States, while the Spanish version (Díaz Rivera et al., 2017) was administered in Mexico.

### 2.3. Procedure

At the beginning of the survey, individuals were informed that the survey was intended to ask questions about health behaviors and how people interact with their environment. Before starting the survey, informed consent was obtained from the participants, indicating that they had read and understood the explanations and were voluntarily participating in the study. In addition, participants were informed that their data would be kept anonymous and confidential. The study and consent procedures were performed in accordance with the ethical standards of the Declaration of Helsinki (1964).

### 2.4. Data analysis

Data were analyzed using the *psych* (Revelle, 2021) and *lavaan* package (Rosseel, 2012) in *R* (R Core Team, 2022). First, descriptive statistics of each item were assessed for the MXINDCOL scale and the INDCOL scale. Then, CFA with Diagonally Weighted Least Squares (DWLS) with a robust method using a polychoric correlation matrix estimation, was used to assess the model fit of the two scales. The DWLS estimation was chosen due to the ordinal observed nature of the variables (Li, 2016), and because of the robustness of the polychoric correlation estimates against moderate violations of normality assumption (Flora and Curran, 2004). The following model fit indices were assessed in the present study: the comparative fit index (CFI), the Tucker-Lewis Index (TLI), the root mean square error of approximation (RMSEA) and its 90% confidence intervals, and the standardized root mean square residual (SRMR). CFI and TLI values above 0.95 are commonly interpreted to indicate excellent model fit, whereas values in the range of 0.90 to 0.95 indicate acceptable fit. The cutoff criteria for excellent fit are 0.08 for SRMR, and 0.6 for RMSEA (Bentler, 1990; Hu and Bentler, 1999; Schweizer, 2010; van de Schoot et al., 2012). Results of the chi-square test (χ^2^) were also reported; however, the chi-square test statistic can be considered unreliable in the context of larger sample sizes (Byrne, 2001).

To assess reliability and convergent and discriminant validity, the composite reliability (CR), maximal reliability [MaxR (H)], average variance extracted (AVE), and square root of average AVE were used. CR and MaxR (H) values above 0.70 were used given that they are commonly interpreted as indicating good reliability. AVE values above 0.50 are interpreted as having a good value; however, this AVE index has been identified as a strict criterion (Malhotra and Dash, 2011; Henseler et al., 2015). In addition, Cronbach’s alpha (α) and McDonald’s Omega (ω) were estimated, as the McDonald’s ω is a better reliability estimate under most conditions than Cronbach’s α (*cf.*, Zinbarg et al., 2006).

Cultural comparisons are commonly studied when trying to understand the way individuals with different backgrounds behave. Comparisons between the English and Spanish-speaking countries are commonly reported, but some of those studies do not assure the same measurement properties in the goal countries. Individualism and collectivism scores were compared between US and Mexican participants. Before evaluating the comparison, cross-cultural measurement invariance across the US and Mexican samples was assessed. Invariance was tested at configural (same structure across groups), metric (same factor loadings across groups), and scalar levels (same item intercepts across groups). These models were compared using Δχ^2^, Δdf, ΔCFI, ΔRMSEA, and ΔSRMR. Based on Chen (2007), when the sample size is ≤300 or when sample sizes are unequal, a change of ≤0.005 in CFI, supplemented by a change of ≤0.010 in RMSEA or a change of ≤0.025 in SRMR, indicates invariance.

## 3. Results

### 3.1. Descriptive statistics

Means, standard deviation, skewness, and kurtosis coefficients were assessed for each item of the MXINDCOL (Table 2).

**Table 2:** Means, standard deviations, skewness, and kurtosis per item of the English and Spanish version of the Mexican vertical and horizontal individualism and collectivism scale (MXINDCOL) in the United States and Mexican samples.

Items	United States					Mexico				
	*M*	*SD*	*CI 95%*	*s*	*k*	*M*	*SD*	*CI 95%*	*s*	*k*
IndCol_1 – I enjoy collaborating with others (Me gusta colaborar con los demás)	4.49	1.63	[4.30, 4.68]	−0.38	−0.51	5.47	1.41	[5.32, 5.63]	−0.96	0.65
IndCol_2 – I collaborate with others to make things work out well (Colaboro con los demás para que las cosas funcionen)	4.79	1.58	[4.51, 4.97]	−0.65	−0.10	5.67	1.21	[5.53, 5.80]	−0.88	0.52
IndCol_3 – I am supportive with my group (Soy solidario con mi grupo)	5.34	1.28	[5.19, 5.48]	−0.96	1.35	5.83	1.25	[5.69, 5.96]	−1.20	1.14
IndCol_4 – I take others into account when making decisions (Tomo en cuenta a los demás en la toma de decisiones)	5.51	1.33	[5.36, 5.67]	−1.18	1.82	5.58	1.27	[5.44, 5.72]	−0.79	0.52
IndCol_5 – I give myself to others without expecting anything in return (Me doy a los demás sin esperar nada a cambio)	5.21	1.41	[5.05, 5.38]	−0.73	0.35	5.30	1.46	[5.14, 5.46]	−0.88	0.58
IndCol_6 – I am a unique individual (Soy un individuo único)	5.50	1.46	[5.33, 5.67]	−0.84	0.35	5.54	1.65	[5.36, 5.72]	−1.22	0.86
IndCol_7 – I am special (Soy especial)	4.64	1.74	[4.44, 4.84]	−0.42	−0.54	4.86	2.12	[4.64, 5.09]	−0.73	−0.79
IndCol_8 – I enjoy being unique and different (Disfruto ser único(a) y diferente)	5.09	1.57	[4.91, 5.27]	−0.53	−0.38	5.42	1.76	[5.22, 5.61]	−1.12	0.43
IndCol_9 – I am original, just as others may be (Soy original como lo pueden ser otros)	5.42	1.39	[5.26, 5.58]	−0.87	0.72	5.74	1.44	[5.59, 5.90]	−1.33	1.56
IndCol_10 – I worry about what others might say (Me preocupa el qué dirán)	3.92	1.82	[3.71, 4.13]	−0.01	−1.03	3.29	1.91	[3.09, 3.50]	0.31	−1.07
IndCol_11 – I feel anxious when others get angry with me (Siento ansiedad cuando los demás se enojan conmigo)	4.82	1.92	[4.60, 5.04]	−0.56	−0.84	3.40	2.05	[3.18, 3.62]	0.32	−1.21
IndCol_12 – My happiness depends on others’ happiness (Mi felicidad depende de la felicidad de los demás)	3.53	1.73	[3.33, 3.73]	0.13	−0.90	2.49	1.63	[2.27, 2.63]	0.97	0.13
IndCol_13 – I prefer not saying anything over making others feel uncomfortable (Prefiero callar que incomodar a los demás)	4.63	1.67	[4.44, 4.82]	−41	−0.60	3.41	1.94	[3.20, 3.62]	0.30	−1.01
IndCol_14 – I am better than others (Soy mejor que los demás)	2.64	1.65	[2.45, 2.83]	0.64	−0.64	2.61	1.81	[2.42, 2.81]	0.85	−0.32
IndCol_15 – I win when I compete because I am good (Cuando compito gano porque soy bueno(a))	3.77	1.65	[3.58, 3.96]	−0.14	−0.76	4.05	1.89	[3.84, 4.25]	−0.14	−1.03
IndCol_16 – I enjoy being in situations that involve competing with others (Disfruto estar en situaciones que implican competir con otros)	3.23	1.70	[3.03, 3.42]	0.32	−0.72	3.53	1.92	[3.32, 3.73]	0.17	−1.10

Note: 95% confidence interval for mean.

### 3.2. Confirmatory factor analysis

**Figure 1:**
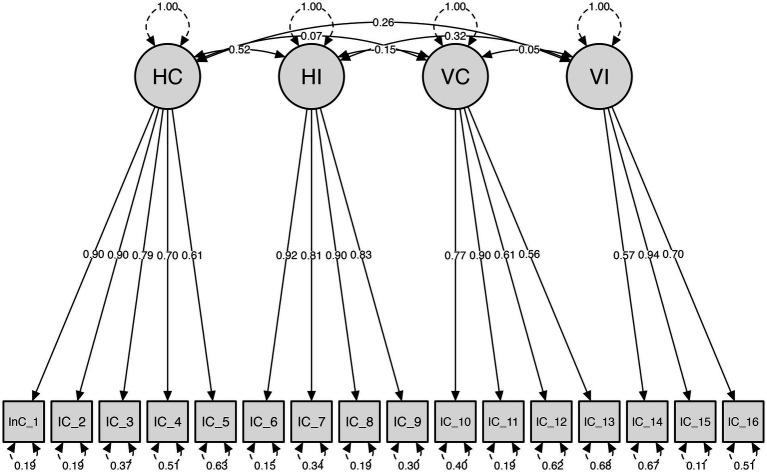
Confirmatory factor analysis of the MXINDCOL scale assessed in the US sample.

To assess the model fit of the individualism and collectivism scales previously reported, CFA was analyzed. First, the structural validity evidence of the MXINDCOL scale in the US (Figure 1), and the Mexican sample through model fit was assessed. Then, the structural validity of the INDCOL in the US and the Mexican sample through model fit was assessed (Table 3). The model fit of the MXINDCOL scale showed a good fit with the data for the US and the Mexican sample. While the RMSEA value in the US sample indicated poor fit, we can turn to the SRMR index as a more suitable parameter for assessing goodness of fit when ordinal data are incorporated into the model (Shi et al., 2019). While the INDCOL for the US sample and the Mexican sample did not show a good fit with the data. These results provide structural validity evidence for the MXINDCOL.

**Table 3:** Model fit for confirmatory factor analysis of the MXINDCOL and the INDCOL scales in the United States and Mexican samples.

Place	MXINDCOL					INDCOL				
	χ^2^	CFI	TLI	RMSEA (90% CI)	SRMR	χ^2^	CFI	TLI	RMSEA (90% CI)	SRMR
United States	376.01	0.981	0.977	0.099 (0.088–109)	0.081	551.18	0.930	0.914	0.126 (0.116–0.136)	0.107
Mexico	202.59	0.993	0.992	0.057 (0.046–0.068)	0.061	583.73	0.936	0.921	0.122 (0.113–0.132)	0.111

### 3.3. Convergent and discriminant validity evidence of the MXINDCOL scale in the US and the Mexican sample

Reliability and convergent and discriminant validity were assessed in the US and the Mexican sample, based on CR, MaxR (H), AVE, and the square root of AVE (Table 4). All scales showed adequate reliability. However, Malhotra and Dash (2011) suggested that AVE is a strict criterion; therefore, CR values are enough to confirm the evidence of convergent validity. The scale was also found to have good discriminant validity across the groups because the square root of AVE was higher than the correlations of the dimensions (Hair et al., 2018).

**Table 4:** Reliability, convergent, and discriminant validity evidence of the MXINDCOL in the United States (*n* = 292) and Mexican sample (*n* = 332).

	CR	AVE	MaxR(H)	Horizontal collectivism	Horizontal individualism	Vertical collectivism	Vertical individualism
United States sample							
Horizontal collectivism	0.86	0.56	0.90	**0.74**			
Horizontal individualism	0.90	0.69	0.91	0.46**	**0.83**		
Vertical collectivism	0.77	0.47	0.84	0.01	−0.13	**0.69**	
Vertical individualism	0.76	0.53	0.88	0.29**	0.30**	−0.01	**0.73**
Mexican sample							
Horizontal collectivism	0.86	0.57	0.90	0.75			
Horizontal individualism	0.86	0.62	0.89	0.40**	**0.79**		
Vertical collectivism	0.77	0.47	0.85	−0.01	−0.08	**0.68**	
Vertical individualism	0.77	0.54	0.82	0.16*	0.38**	−0.02	**0.73**

^a^Values in the diagonal represent the square root of AVE. **p < 0.001, *p < 0.05.

Cronbach’s alpha, McDonald’s omega, kurtosis, and skewness coefficients of the two individualism and collectivism scales were assessed in the US and Mexican samples (Table 5). The MXINDCOL scale, evaluated in both the US and Mexican samples, demonstrated superior reliability across all factors compared to the INDCOL scale.

**Table 5:** Cronbach’s Alpha, McDonald’s Omega, kurtosis, and skewness coefficients.

	United States				Mexico			
	α	ω	*s*	*k*	α	ω	*s*	*k*
MXINDCOL								
Horizontal collectivism	0.86	0.86	−0.74	0.82	0.86	0.85	−0.74	0.36
Horizontal individualism	0.89	0.89	−0.70	0.28	0.85	0.85	−0.95	0.36
Vertical collectivism	0.77	0.78	−0.32	−0.36	0.75	0.77	0.34	−0.55
Vertical individualism	0.74	0.76	0.26	−0.34	0.76	0.77	0.23	−0.67
INDCOL								
Horizontal collectivism	0.79	0.79	−0.88	1.07	0.69	0.69	−0.66	−0.27
Horizontal individualism	0.73	0.75	0.36	−0.16	0.75	77.	0.45	−0.44
Vertical collectivism	0.79	0.79	−0.61	0.98	0.75	0.74	−0.72	0.15
Vertical individualism	0.78	0.80	−0.52	0.18	0.74	0.82	−0.10	−1.05

Note: United States sample, *n* = 292; Mexico sample, *n* = 332. *α* = Cronbach’s alpha, *ω* = McDonald’s omega, *k* = kurtosis, *s* = skewness.

### 3.4. Cross-cultural measurement invariance

The measurement model of the MXINDCOL was assessed based on country invariance. For this, the configural, metric, and scalar invariance were evaluated using multigroup modeling (Table 6). First, the configural model assessed in M0 indicates that MXINDCOL measures the same constructs across groups. As configural invariance holds, the model fit of M1 was assessed, showing good model fit and that all factor loadings were equivalent across samples. Therefore, M2 was assessed, indices showed that the hypothesis of item intercept invariance was supported. Derived from these findings, it can be asserted that variations linked to specific countries, discernible through the scores acquired from the assessment instrument, do not arise due to any shortcomings in the measurement process. Thus, this procedure ensures configural, metric, and scalar invariance. Overall, strong invariance indicators were determined according to Widaman and Reise (1997).

**Table 6:** Testing for factorial invariance of the MXINDCOL scale across cultures, United States (*n* = 292) and Mexico (*n* = 332) groups.

Model	*χ^2^*	*df*	*CFI*	*RMSEA* (90% CI)	*SRMR*	Model comparison	Δ*χ^2^*	Δ*df*	Δ*CFI*	Δ*RMSEA*	Δ*SRMR*
(M0)	578.59^**^	196	0.988	0.079 (0.072–0.087)	0.070	–	–	–	–	–	–
(M1)	619.65^**^	208	0.987	080 (0.073–0.087)	0.072	M0 – M1	41.06	12	0.001	0.001	0.002
(M2)	795.13^**^	284	0.983	0.076 (0.070–0.082)	0.071	M1 – M2	175.48	76	0.004	0.004	0.004

^a^M0 = configural, ^b^M1 = metric, ^c^M2 = scalar. * *p* < 0.05, ** *p* < 0.001.

In the present study, cross-cultural comparisons were examined in terms of the origin country. Statistically significant differences were found in terms of horizontal collectivism, horizontal individualism, and vertical collectivism (Table 7). On the one hand, Mexican participants showed higher horizontal collectivism and horizontal individualism than US participants. Conversely, US participants showed higher vertical collectivism than Mexican participants.

**Table 7:** Comparison of MXINDCOL between the United States and México groups.

	United States	Mexico	*W*	value of *p*	Rank-Biseral correlation	Confidence interval 95% for Rank-Biseral correlation
	*M (SD)*	*M (SD)*				
Horizontal collectivism	5.06 (1.16)	5.56 (1.06)	60855.00	< 0.001	0.255	[0.16, 0.33]
Horizontal individualism	5.16 (1.34)	5.39 (1.46)	54582.00	0.006	0.126	[0.03, 0.21]
Vertical collectivism	4.22 (1.38)	3.13 (1.44)	28135.00	< 0.001	−0.420	[−0.49, −0.34]
Vertical individualism	3.21 (1.36)	3.39 (1.55)	51493.50	0.178	0.062	[−0.02, 0.15]

## 4. Discussion

The primary objective of this investigation was to linguistically translate and culturally adapt the MXINDCOL scale into English. This measurement tool exhibits a theoretically consistent conceptual framework, underscored by substantiating adequate validity and reliability indicators. Furthermore, conceptual alignment exists between this scale and the assessment of cultural orientation according to Triandis and Gelfand’s instrument from 1998. Additionally, the structural configuration of the English version of the MXINDCOL scale, as established in this study, aligns with the structural pattern previously observed in the Mexican population by Soler-Anguiano and Díaz-Loving (2017), as well as by Díaz-Loving et al. (2018).

Although the previously developed measurement of individualism and collectivism (Triandis and Gelfand, 1998) has moderate psychometric properties, its stability and psychometric properties are distorted when translated to Spanish-speaking regions (see Díaz Rivera et al., 2017). The individualism and collectivism scale translated and adapted in the present study provides evidence that the cross-cultural adequation of the construct is more helpful in identifying elements belonging to the construct. These results suggest that cultural aspects can be involved in understanding the construct and shaping behaviors in certain regions.

In this study, the CFA revealed a significantly better fit for the MXINDCOL scale compared to the INDCOL scale. This highlights the presentation of a scale primarily comprising personality traits across its entirety. While it is recommended to explore norms and beliefs when assessing individualism and collectivism at the individual level, caution must be exercised to prevent mixed orientations within the scale. These findings underscore the importance of creating scales with careful consideration of potential item biases (*cf.* Van de Vijver and Poortinga, 1997). On the other hand, the adequate fit of the models may be attributed to the inclusion of local elements not previously identified, emphasizing one of the advantages of conducting cultural assessments in contexts different from the ones originally examined. This practice enables us to differentiate between differences arising from ethnocentric bias and those inherent to the two cultures (Campbell, 1970).

The English version of the MXINDCOL showed minor issues when displaying convergent validity evidence. The AVE for the vertical collectivism dimension did not meet the strict criterion of convergent validity evidence. However, previous literature suggests relying on other indices to ensure evidence of convergent validity, as proposed by Malhotra and Dash (2011). The discriminant validity was completely attained.

We want to point out that coefficient α reliability values were above the recommended 0.70 for the dimensions compared with the reliability values of the INDCOL in our study. This finding supports the use of the translated and adapted version made in Mexico. Possibly, the items in the MXINDCOL scale measure updated issues assessed by the INDCOL scale in the 25 years since it was developed. Owing to the nature of the construct, it is essential to have a scale that can be used in different cultural contexts. Therefore, the MXINDCOL scale translated and adapted in the present study was invariant across the US and Mexican samples. In other words, participants from the US and Mexico in the present study perceived individualism and collectivism constructs similarly. With this, differences in factor scores could not be attributed to differences in understanding of the construct. The invariant property of the scale allowed us to compare these two cultures.

The present research has pointed out the differential way to interiorize hierarchical elements, specifically with the item “*I prefer not saying anything over making others feel uncomfortable,”* US participants showed a higher mean than the Mexican sample. This element has typically been defined as abnegation in some studies in cultures like Mexico (Díaz-Guerrero, 1994), where reference group members’ well-being is more important than one’s own. This finding can support the previous evidence of the reconfiguration made by Li and Aksoy (2007), re-specifying the model of Triandis and Gelfand (1998). Both pieces of evidence may suggest that even when a scale includes what appears to be a distinctive element of only one culture, individualists and collectivists can display similar ways of interacting with the cultural background. One might attribute it to age differences, but no differences in age were found through the different MXINDCOL dimensions. It can suggest that tackling the culture assessment by including typically classified idiosyncratic elements can enrich understanding of the construct and its parts.

The present findings may contribute to the development of culturally sensitive psychometric scales by tackling measurement biases (Van de Vijver and Poortinga, 1997) and following a bottom-up perspective when assessing a construct (Cheung et al., 2011). Future studies are needed to cross-culturally assess these constructs and identify those idiosyncratic and universal elements that increase understanding of cultural orientations. As a rule, if there is suspicion that the samples are different on some demographic variables, these variables should be measured and their impact assessed (Van de Vijver and Poortinga, 1997).

The MXINDCOL scale has shown good model fit and measurement invariance through the US and Mexican samples. Once we had a scale that showed to be invariant across the two samples, comparisons of MXINDCOL dimensions were assessed. From this, it was noticed that horizontal dimensions were the ones that presented higher scores in the Mexican sample compared with the US sample. These findings might reflect consistency with previous research, highlighting the egalitarian orientation of the Mexican population (Díaz-Guerrero, 1994; Triandis, 2000, 2006; Hofstede, 2001; Chen and West, 2008). Conversely, the US sample displayed a higher vertical collectivism score than the Mexican sample. These results are suggested with caution because it has been consistently shown that Mexicans are collectivist at a cultural level, but the higher the academic level, the closer to individualistic representations (Díaz-Loving, 1998). The academic level should be an element to consider for future studies.

Some additional limitations related to this research are bonded to the sample. We sacrificed the sample size to have the most equivalent samples possible so they can be comparable. The sample at the beginning was unequal in terms of demographic characteristics. We selected the propensity score matching to counterbalance confounding effects as suggested by Rosenbaum and Rubin (1983, 1985) to balance by age and sex. This procedure significantly reduced confounder effects but increased the consequences derived from sample sizes. Despite having found no significant differences in estimates when running the analysis with the sample before and after the propensity score matching, it is suggested to increase the sample and assess CFA estimates in different samples. Thus, replication studies with different samples are highly recommended for future research, caring for selecting and balancing equivalent cultural groups.

In summary, the MXINDCOL scale can be considered an easy-to-use and reliable tool for assessing collectivism and individualism. Evidence of the suitability of the scale in more populations might support the understanding of cultural dimensions by incorporating idiosyncratic elements and refining the item structure within the scale. This research might apport the understanding of cultural dimensions by incorporating idiosyncratic elements and refining the item structure within the scale. Hence, the MXINDCOL scale could do this effectively.

## Data availability statement

The datasets generated and analysed for this study can be found in the Open Science Framework repository at https://osf.io/bpcfg/?view_only=dd98f5e30409477985c1c037c18c24c1.

## Ethics statement

The studies involving humans were approved by the National Autonomous University of México. The studies were conducted in accordance with the local legislation and institutional requirements. The participants provided their written informed consent to participate in this study.

## Author contributions

All authors contributed to the study’s conception and design. Material preparation, data collection, and analysis were performed by FS-A. The first draft of the manuscript was written by FS-A, and all authors commented on previous versions of the manuscript. All authors contributed to the article and approved the submitted version.

## 5. Appendix

The vertical and horizontal individualism and collectivism scale (INDCOL) and the Mexican vertical and horizontal individualism and collectivism scale (MXINDCOL).

**Table:**

INDCOL (Triandis and Gelfand, 1998)	MXINDCOL (Díaz-Loving et al., 2018)
*Horizontal Collectivism*	
1. If a coworker gets a prize, I would feel proud	1. I enjoy collaborating with others
2. The well-being of my coworkers is important to me	2. I collaborate with others to make things work out well
3. To me, pleasure is spending time with others	3. I am supportive with my group
4. I feel good when I cooperate with others	4. I take others into account when making decisions
	5. I give myself to others without expecting anything in return
*Horizontal individualism*	
5. I’d rather depend on myself than others	6. I am a unique individual
6. I rely on myself most of the time; I rarely rely on others	7. I am special
7. I often do “*my own thing*”	8. I enjoy being unique and different
8. My personal identity, independent of others, is very important to me	9. I am original, just as others may be
*Vertical Collectivism*	
9. Parents and children must stay together as much as possible.	10. I worry about what others might say
10. It is my duty to take care of my family, even when 1 have to sacrifice what I want	11. I feel anxious when others get angry with me
11. Family members should stick together, no matter what sacrifices are required.	12. My happiness depends on others’ happiness
12. It is important to me that I respect the decisions made by my groups	13. I prefer not saying anything over making others feel uncomfortable
*Vertical Individualism*	
13. It is important that I do my job better than others	14. I am better than others
14. Winning is everything	15. I win when I compete because I am good
15. Competition is the law of nature.	16. I enjoy being in situations that involve competing with others
16. When another person does better than I do, I get tense and aroused.	
