# Supplementary material for: Cross-cultural measurement invariance evidence of individualism and collectivism: from the idiosyncratic to universal
Source: Front Psychol. 2023 Sep 27;14:1150757. doi: 10.3389/fpsyg.2023.1150757 (PMC10565350; doi:10.3389/fpsyg.2023.1150757)
Supplement: Supplementary file 1 [file Table_1.pdf]

Parameter estimates for Confirmatory Factor Analysis of the MXINDCOL and INDCOL scales assessed in the USA and Mexico

USA - MXINDCOL
Factor loadings

						95% Confidence Interval	
**Factor**	**Indicator**	**Estimate**	**Std. Error**	**z-value**	**p**	**Lower**	**Upper**
HC	IndCol_1	0.903	0.016	56.640	< .001	0.871	0.934
	IndCol_2	0.901	0.017	54.088	< .001	0.869	0.934
	IndCol_3	0.793	0.023	34.886	< .001	0.748	0.837
	IndCol_4	0.703	0.032	21.785	< .001	0.640	0.767
	IndCol_5	0.611	0.040	15.344	< .001	0.533	0.689
HI	IndCol_6	0.921	0.013	70.981	< .001	0.895	0.946
	IndCol_7	0.811	0.021	38.518	< .001	0.770	0.852
	IndCol_8	0.898	0.015	58.905	< .001	0.868	0.928
	IndCol_9	0.834	0.021	40.012	< .001	0.794	0.875
VC	IndCol_10	0.775	0.027	28.758	< .001	0.722	0.828
	IndCol_11	0.900	0.024	37.542	< .001	0.853	0.947
	IndCol_12	0.613	0.040	15.212	< .001	0.534	0.692
	IndCol_13	0.563	0.042	13.420	< .001	0.481	0.645
VI	IndCol_14	0.574	0.046	12.574	< .001	0.484	0.663
	IndCol_15	0.943	0.047	20.162	< .001	0.852	1.035
	IndCol_16	0.701	0.045	15.532	< .001	0.613	0.790

Mexico – MXINDCOL
Factor loadings

						95% Confidence Interval	
**Factor**	**Indicator**	**Estimate**	**Std. Error**	**z-value**	**p**	**Lower**	**Upper**
HC	IndCol_1	0.860	0.018	48.201	< .001	0.825	0.895
	IndCol_2	0.932	0.010	89.866	< .001	0.912	0.953
	IndCol_3	0.855	0.018	47.491	< .001	0.820	0.890
	IndCol_4	0.728	0.028	25.748	< .001	0.673	0.784
	IndCol_5	0.636	0.030	21.425	< .001	0.578	0.694
HI	IndCol_6	0.850	0.020	43.442	< .001	0.811	0.888
	IndCol_7	0.731	0.028	26.055	< .001	0.676	0.785
	IndCol_8	0.923	0.015	61.404	< .001	0.894	0.953
	IndCol_9	0.866	0.019	45.295	< .001	0.828	0.903
VC	IndCol_10	0.688	0.034	20.528	< .001	0.622	0.754
	IndCol_11	0.921	0.023	39.811	< .001	0.876	0.967
	IndCol_12	0.762	0.031	24.330	< .001	0.701	0.824
	IndCol_13	0.491	0.044	11.114	< .001	0.405	0.578
VI	IndCol_14	0.584	0.043	13.506	< .001	0.499	0.669
	IndCol_15	0.948	0.041	22.945	< .001	0.867	1.029
	IndCol_16	0.743	0.040	18.624	< .001	0.665	0.821

USA- INDCOL
Factor loadings

						95% Confidence Interval	
**Factor**	**Indicator**	**Estimate**	**Std. Error**	**z-value**	**p**	**Lower**	**Upper**
HC	IndColTriandis_1	0.800	0.030	26.810	< .001	0.742	0.859
	IndColTriandis_2	0.794	0.030	26.279	< .001	0.735	0.853
	IndColTriandis_3	0.833	0.026	31.483	< .001	0.781	0.885
	IndColTriandis_4	0.633	0.040	15.706	< .001	0.554	0.712
HI	IndColTriandis_5	0.558	0.044	12.726	< .001	0.472	0.644
	IndColTriandis_6	0.930	0.044	21.111	< .001	0.844	1.017
	IndColTriandis_7	0.714	0.044	16.156	< .001	0.628	0.801
	IndColTriandis_8	0.569	0.047	12.056	< .001	0.477	0.662
VC	IndColTriandis_9	0.703	0.034	20.470	< .001	0.636	0.771
	IndColTriandis_10	0.824	0.027	30.971	< .001	0.772	0.876
	IndColTriandis_11	0.679	0.036	18.787	< .001	0.608	0.750
	IndColTriandis_12	0.839	0.024	35.642	< .001	0.792	0.885
VI	IndColTriandis_13	0.704	0.034	20.650	< .001	0.637	0.771
	IndColTriandis_14	0.713	0.046	15.601	< .001	0.624	0.803
	IndColTriandis_15	0.818	0.027	30.547	< .001	0.766	0.871
	IndColTriandis_16	0.852	0.024	36.260	< .001	0.806	0.898

Mexico – INDCOL
Factor loadings

						95% Confidence Interval	
**Factor**	**Indicator**	**Estimate**	**Std. Error**	**z-value**	**p**	**Lower**	**Upper**
HC	IndColTriandis_1	0.721	0.044	16.309	< .001	0.634	0.808
	IndColTriandis_2	0.826	0.038	21.582	< .001	0.751	0.902
	IndColTriandis_3	0.726	0.039	18.418	< .001	0.649	0.804
	IndColTriandis_4	0.579	0.049	11.864	< .001	0.484	0.675
HI	IndColTriandis_5	0.687	0.042	16.484	< .001	0.605	0.768
	IndColTriandis_6	0.852	0.028	29.974	< .001	0.796	0.907
	IndColTriandis_7	0.806	0.032	24.903	< .001	0.743	0.870
	IndColTriandis_8	0.553	0.045	12.251	< .001	0.465	0.641
VC	IndColTriandis_9	0.830	0.028	29.248	< .001	0.774	0.885
	IndColTriandis_10	0.913	0.023	38.967	< .001	0.867	0.958
	IndColTriandis_11	0.567	0.039	14.404	< .001	0.490	0.644
	IndColTriandis_12	0.754	0.034	21.939	< .001	0.687	0.821
VI	IndColTriandis_13	0.710	0.031	22.930	< .001	0.650	0.771
	IndColTriandis_14	0.437	0.050	8.733	< .001	0.339	0.535
	IndColTriandis_15	0.926	0.024	37.923	< .001	0.879	0.974
	IndColTriandis_16	0.879	0.026	33.508	< .001	0.827	0.930
